# Depressive Symptoms and Emotional Distress of Transnational Mothers: A Scoping Review

**DOI:** 10.3389/fpsyt.2021.574100

**Published:** 2021-02-25

**Authors:** María Pineros-Leano, Laura Yao, Aroub Yousuf, Gabrielle Oliveira

**Affiliations:** ^1^School of Social Work, Boston College, Chestnut Hill, MA, United States; ^2^Philosophy Department, Boston College, Chestnut Hill, MA, United States; ^3^Teaching, Curriculum, and Society, Lynch School of Education and Human Development, Boston College, Chestnut Hill, MA, United States

**Keywords:** transnational mother, immigration, mental health, emotional distress, depressive symptoms, maternal depression

## Abstract

**Background:** Female led migration is a recent trend that has been gaining momentum, particularly in Latin America. However, little attention has been given to the psychological consequences of mothers who leave their children in their country of origin and migrate to a host country to work. Therefore, it is important to investigate the mental health status of transnational mothers and to further identify issues for intervention and supportive services.

**Methods:** PubMed, PsycINFO, ERIC, CENTRAL, Scopus, and ScienceDirect databases were searched systematically for peer-reviewed articles published from inception through July 2019. The search included the following terms: migrant, immigrant, transnational, transnational mother, AND mood disorders, depressive symptoms, and depression. Initially, 8,375 studies were identified. After exclusionary criteria were applied, 17 studies were identified and included in the review.

**Results:** We found six quantitative studies that investigated depressive symptoms among transnational mothers. Of these studies, three found a positive association between transnational motherhood and depressive symptoms; three of these articles found a null correlation. A total of eight qualitative studies and three mixed-methods studies were found that addressed depressive symptoms and emotional distress among transnational mothers. The eight qualitative studies identified highlighted the significant emotional distress transnational mothers experience. Lastly, the three mixed-methods studies similarly discussed the emotional hardships faced by transnational mothers.

**Implications:** The studies identified suggest that depressive symptoms and emotional distress are prevalent among transnational mothers. Therefore, public health social workers and other mental health providers need to focus on developing strategies to identify and screen transnational mothers for depressive symptoms.

## Introduction

International migration was historically led by males (1). However, in recent decades, female migration has increased (1). Recent data suggest that 47% of international migrants around the globe are female (2). In the United States (U.S.), 51.7% of immigrants are women (3). From 1990-2017, the percent of female migrants has increased in most regions around the globe, with the exception of East Asia and the Pacific, possibly due to an increased demand in male-dominated work (4). Female migration patterns appear to fluctuate due to various economic and political forces within each country and the feminization of labor (4). However, at a population level, it is currently unknown how many of these women are mothers and are separated from their children.

Transnational motherhood, a term coined initially by Hondagneu-Sotelo (5), constitutes a phenomenon in which mothers are forced to leave their children in their country of origin in order to be able to work in the host country. Investigating transnational motherhood is important for three reasons. First, mothers are often key attachment figures for children (6, 7). As key attachment figures, the initial relationships children have with their mothers impacts subsequent relationships (6–8). Moreover, the rupture of this bond, as can occur when mothers migrate apart from their children, can have lasting harmful effects on children (9, 10). A second reason it is important to focus on transnational mothers has to do with the role and the expectations that women have around motherhood. In many cultures, motherhood is often a central part of women's identity. From a social constructivist perspective, motherhood is often heavily influenced by gender norms that portray women as self-sacrificing and emotionally bound to their children (11). For transnational mothers, emotional intimacy can feel compromised by distance, and women can fall short of social expectations, which can contribute to women's feelings of failure (11). The third reason investigating transnational motherhood is important is because we still have limited research available in this population. For instance, we do not know how prevalent transnational motherhood is, the characteristics of the mothers who migrate, or the situation in which mothers live post-migration.

Immigrants from Mexico and Latin American countries make up 50% of the overall immigrant population in the U.S., making Latin America the largest region where immigrants come from (3). Although women migrate for various reasons, many Latin American women (specifically from El Salvador, Honduras, and Guatemala) migrate to the U.S. due to political strife and economic instability in their countries of origin (12). In a study of 57 Latina transnational mothers, the participants characterized their migration as a sacrifice made for the well-being of their children (5). For a majority of transnational mothers, motherhood is continuously identified as their primary identifying factor over wifehood or employment (13). However, because of financial constraints, transnational mothers often feel that it is their duty to work abroad to fund their children's education and other needs (14). Leaving children in the country of origin complicates the migratory process given that many immigrant women may already have to deal with all of the hardships of migration (low-wage jobs, poverty, isolation, and discrimination, among others) plus the emotional burden of not being physically with their children (15, 16). The emotional tumult is exacerbated by legal uncertainty propagated through immigration policy (12).

Contextual experiences in the countries transnational mothers migrate to also play a role in the emotional well-being of these mothers. For instance, negative social discourse around immigration and restrictive immigration laws have been linked to depression and anxiety among immigrants (17). In the United States, for instance, state-level immigration policies that are restrictive for the Latinx population have been linked to depression, anxiety, stress, feelings of isolation, and lowered self-esteem among Latinx individuals who reside in these states (18). As anti-immigrant rhetoric has escalated in several countries in recent years, depression and emotional distress among immigrants appear to have exacerbated (12).

Depression affects over 300 million people around the world and it is the most prevalent mental health condition worldwide (19). Depression has been found to be twice as prevalent among women than men across different societies (20). Studies available on depression and depressive symptoms among immigrant populations suggests they have lower levels of depressive symptoms, compared to their native-born counterparts (19, 20). However, the case might be different for mothers who are separated from their children. To date, most research available on transnational families explores the sequelae that separation has on the children who stay in the country of origin (21–24). For instance, a systematic review and meta-analysis investigated the effects of parental migration on the health and mental health of children (21). The review summarized information from 111 studies and found that children who had stayed in the country of origin experienced an increased risk of negative mental health outcomes such as depressive symptoms, anxiety, and suicidal ideation, among others (21). Although an abundant amount of scientific evidence suggests that family separation due to immigration has a negative impact on children's mental health, the effects that transnational motherhood has on the mental health of mothers are not clear. To address this gap, the purpose of this study was to provide a landscape of quantitative, qualitative, and mixed methods studies that have investigated the emotional sequelae of transnational motherhood on immigrant mothers, particularly around depressive symptoms.

## Methods

This study utilized Arskey and O'Malley's methodological framework for conducting scoping reviews (25) since it is the recommended framework for these type of reviews (26). A scoping review was chosen given that the literature among transnational mothers is still in its early stages; therefore, it was pertinent to provide an overview of the available research to better understand this emerging field (27). In accordance with Arskey and O'Malley's framework, the review consisted of five stages, which included (1) identifying the research question, (2) identifying relevant studies, (3) selecting studies, (4) charting the data, and (5) collating, summarizing and reporting the results (25).

### Identifying the Research Question

Before the search was conducted, we identified the research question. Leaving children behind in the country of origin is no easy task. For women, this task may be more difficult since historically they have been the ones in charge of children's care and emotional well-being (28). The research question guiding this study is: what are the emotional and mental health needs of immigrant mothers who leave their children in their country of origin?

### Identifying Relevant Studies

The search was conducted using electronic searches in the following databases: PubMed, PsycINFO, ERIC, CENTRAL, Scopus, and ScienceDirect. The search included the following terms: women, migrant, immigrant, transnational, transnational mother, immigrant mother, migrant mother, AND mood disorders, emotional distress, and depression (Supplementary Table 1).

Researchers created the search terms to most adequately identify relevant studies. The inclusion criteria included any studies that were conducted on: (1) immigrant mothers, (2) who had at least one child in the country of origin, and (3) discussed emotional distress or mental health, particularly as it pertained to depressive symptoms (9). We did not specify countries of origin or destination for participants in an effort to encompass as much information as possible. We did not rule out studies that focused on both parents; however, our results focus strictly on the experiences of mothers.

### Study Selection

**Figure 1:**
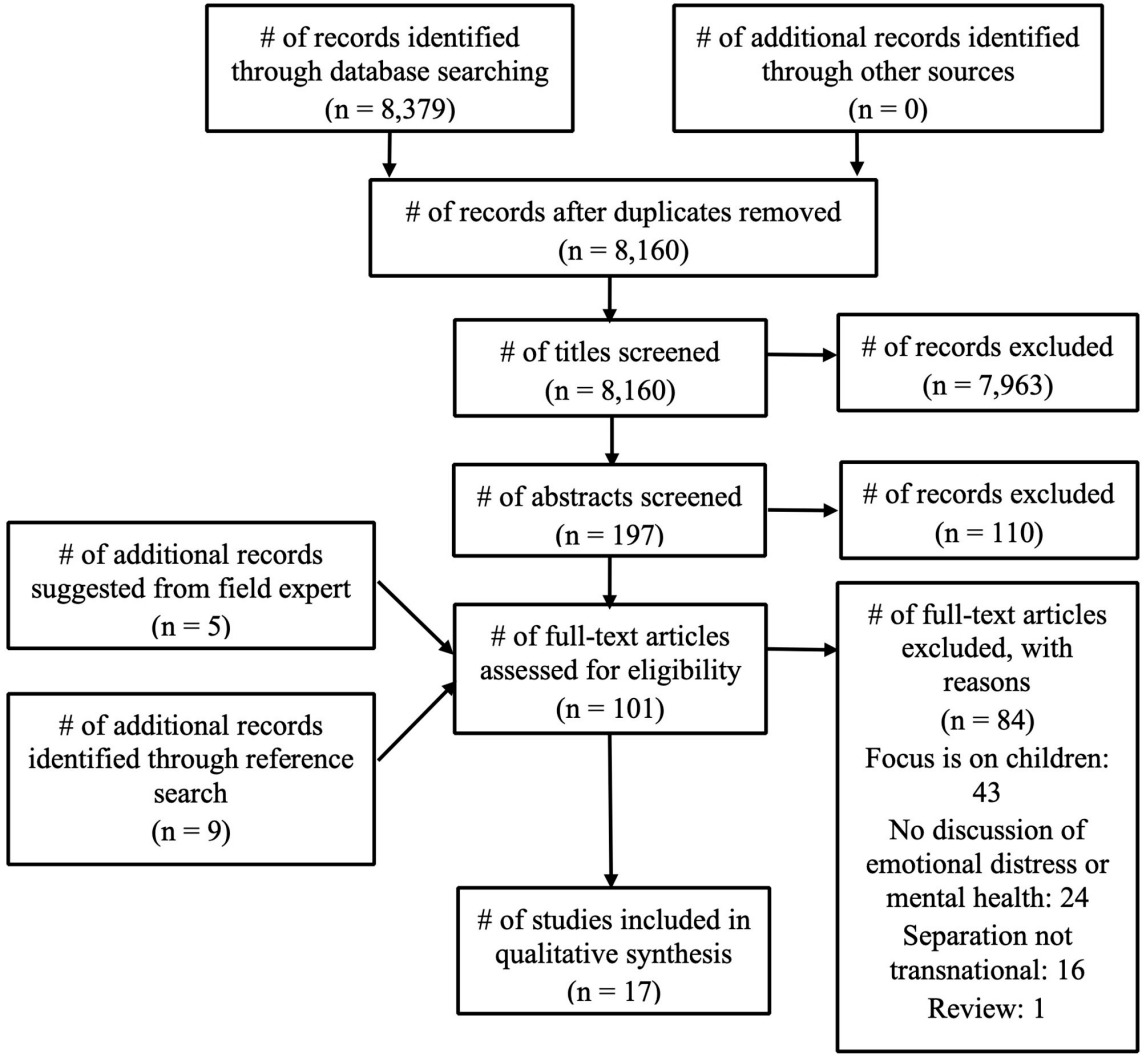
Flow chart of study selection.

The initial search yielded 8,160 articles after duplicates were removed (Figure 1). One researcher reviewed all the titles and identified 197 potential matches that fit the inclusion criteria. Two researchers separately reviewed the abstracts of these 197 potential matches and identified 87 articles for full text review. To ensure that all potential articles were being included in the scoping review and to ensure that different disciplines were being considered, the 87 articles were shared with an expert in the field of transnational motherhood. The expert and co-author went through the list of articles and suggested five additional articles that are considered to be in-depth ethnographic studies in the fields of sociology and anthropology. One researcher then went through each article and conducted a reference search, identifying nine additional studies for a total of 101. Of these 101 full-text articles reviewed, two researchers identified 17 studies that met the full inclusion criteria. The studies at this stage were excluded because: (a) they focused exclusively on children's outcomes (*n* = 43); (b) they did not include a discussion of depression, depressive symptoms, or emotional distress (*n* = 24); (c) the separation between mother and child was not transnational (*n* = 16); (d) the study was a review of the effects that transnational separation had on children's mental health (*n* = 1). We included one mixed-methods study that particularly focused on refugees given that some populations would fall under this category and potentially consider themselves refugees without having this legal designation. However, we ruled out studies that focused on separations through deportations, as these are usually forceful and happen in an abrupt manner. The final number of studies included in this scoping review was 17. Quantitative, qualitative, and mixed-methods studies were included.

### Charting and Summarizing the Data

In order to chart and summarize the data available from different studies, we created two separate tables, one for quantitative studies and another for mixed-methods studies. These tables include relevant information about the study including the country of origin of participants, the host country, the sample size, the number of years residing in host country, the number of years separated from children, the tools used to measure depressive symptoms, and the main findings of the study. Table 1 summarizes the information available from quantitative studies. Table 2 summarizes the information available from mixed-methods studies and Table 3 includes information from qualitative studies.

**Table 1:** Characteristics of quantitative studies.

**References**	**Country/countries of origin**	**Host country**	**Sample size**	**Years of residency in host country**	**Number of years separated from children**	**Depression scale**	**Results**
Bouris et al. (29)	Various countries across globe. Regions where transnational mothers came from (%): Africa (33.7), Asia (20.7), Europe and North America (2.2%), and Latin America (43.5)	Canada	*N* = 514. Separated from children *n* = 92	52.2% of transnational mothers had lived in Canada for less than 2 years	N/A	Edinburgh Postnatal Depression Scale (EPDS)	Mothers who were separated from their children had a significantly higher prevalence of elevated depressive symptoms (28.3%), compared to mothers who were not separated from their children (18.6%). Mothers who were separated from their children, also displayed significantly higher symptoms of anxiety (16.5%) and clinical depression related to trauma (23.1%), compared to mothers who were not separated from their children (9.4 and 13.5%, respectively).
McCabe et al. (30).	Various Latin American countries+	USA	*N* = 425. Women with at least one minor child living abroad *n* = 37	Women with at least one minor child *M* = 6.41 (SD = 5.94)	Women with at least one minor child *M* = 5.73 years (SD = 5.47 years)	Center for Epidemiologic Studies Scale (CES-D)	Twenty-four percent of women who had not been separated from their children had elevated depressive symptoms. Twenty-eight percent of women who were separated from an adult child and 24% of women who were separated from a minor child had elevated depressive symptoms. There were not statistically significant differences on elevated depressive symptoms between women who had never been separated from their children, women who were separated from adult children, and women who were separated from minor children.
Miranda et al. (16)	Central America (%): El Salvador (40.9), Nicaragua (13.5), Guatemala (9.4), Mexico (5.6), Belize, Costa Rica, or Honduras (2.5). South America (25.2%)+, and the Caribbean (2.9%)+	USA	*N* = 5,122 Women separated from children *n* = 232	Women separated from their children *M* = 4.1 years (SD = 3.5)	N/A	Primary Care Evaluation of Mental Disorders (Prime-MD)	Among women separated from their children, the prevalence of depression was 18.1%, compared to 11.4% of women who were not separated from their children and 10.9% of women who did not have children. Women who were separated from their children were 1.5 times more likely to have depression.
Ornelas and Perreira (31)	Mexico (78%), El Salvador (4%), Honduras (6%), Central America+ (3%), South America+ (6%), and Caribbean countries+ (3%)	USA	*N* = 281. Women *n* = 223. Separated from children *n* = 124	*M* = 8 years	*M* = 3 years	Center for Epidemiologic Studies Scale (CES-D) and Patient Health Questionnaire (PHQ-9)	The prevalence of depressive symptoms was 26% measured by the PHQ-9 and of substantial depressive symptoms was 14% measured by the CES-D. Reunifying with family was associated with decreased odds of depressive symptoms (measured by the PHQ-9).
Pannetier et al. (32)	Various Sub-Saharan African countries+	France	*N* = 2,442 Women *n* = 1,189 Women with at least one minor child living abroad *n* = 114	Median = 13 years	N/A	Patient Health Questionnaire-4 (PHQ-4)	Twenty-four percent of women reported elevated depressive symptoms. There was not an association between transnational motherhood and elevated depressive/anxiety symptoms, after adjusting for sociodemographic variables.
Rusch and Reyes (33)	Mexico	USA	*N* = 53 Women separated from children *n* = 37	Most participants (*n* = 36) had lived in the US for 10 years or more	*M* = 3.27 years (SD = 1.95 years)	Center for Epidemiologic Studies Scale (CES-D)	Forty-six percent of participants had elevated depressive symptoms. Depressive symptoms of participants who had been separated from their children was not significantly different from participants who had not been separated from their children.

*N/A indicates information was not available. +Countries of origin were not specified*.

**Table 2:** Characteristics of mixed-methods studies.

**References**	**Country/countries of origin**	**Host country**	**Sample size**	**Years of residency in host country**	**Number of years separated from children**	**Depression scale**	**Findings**
Rousseau et al. (34).	Latin America & Africa+	Canada	N = 113	Latin American participants *M* = 54 months African participants *M* = 40 months	*M* = 3.5 years	Symptom Checklist SCL-90R	Quantitative results demonstrated that there was not a statically significant difference in mental health symptoms among participants based on whether they had reunified with their children or not. Qualitative findings demonstrated that parents are worried about their children in their country of origin and want to reunite as soon as possible.
Suarez-Orozco et al. (35)	China (24%), Central America (19%), Dominican Republic (18%), Haiti (16%), and Mexico (23%)	USA	Quantitative longitudinal data *N* = 282 adolescents Qualitative data from parent-adolescent dyads *N* = 309	N/A	*M* = 1.2 years (SD = 1.1 year) 45% of the sample was not separated from the mother, 13% was separated for less than 2 years, 12% between 2 and 4 years, and 26% for 4 or more years	26-item psychological symptom scale developed by the research team. It included 5 domains: depression, anxiety, cognitive functioning, interpersonal sensitivity, and hostility.	Quantitative results only report children's depressive symptomology. Qualitative findings reported that separating from children generated angst and pain on the parents. Mothers maintained regular contact with their children abroad through phone calls, remittances, and letters. The reunification process was particularly difficult when the reunification process took too long; parents had to build trust and authority with their children once reunited.
Suárez-Orozco et al. (36)	China, Central America, Dominican Republic, Haiti, & Mexico	USA	*N* = 385 parent-adolescent dyads	N/A	34% of the sample was separated for their mothers less than 2 years, 38% between 2 and 4 years, and 28% for 5 or more years	26-item psychological symptom scale developed by the research team. It included 5 domains: depression, anxiety, cognitive functioning, interpersonal sensitivity, and hostility.	Quantitative results only report children's depressive symptomology. Qualitative findings suggested that parents had strong feelings of sadness about separating from their children. The findings also suggested that reunification was a complicated process fill of ambivalent emotions and it took time to readjust family dynamics.

*N/A indicates information was not available. +Countries of origin were not specified*.

**Table 3:** Characteristics of qualitative studies.

**Authors**	**Country/countries of origin**	**Host country**	**Sample size**	**Years of residency in host country**	**Number of years separated from children**	**Findings**
Bernhard et al. (37)	Colombia (25%), Costa Rica (15%), Guatemala (12.5%), El Salvador (17.5%), Ecuador (15%), and Mexico (15%)	Canada	*N* = 40	N/A	Separations ranged from 2 to 96 months	Most mothers (*n* = 29) described feeling sad constantly. A total of 15 mothers described feeling very depressed. There were also discussions of feeling guilty for not being with their children. Separations were seen by the mothers as a violation of a cultural norm.
Dreby (11)	Mexico	USA	*N* = 43 Mothers n = 21	*M* = 3.4	Length of separation from mothers *M* = 3.4 years	Findings indicated that they suffered greatly because they were not with their children. In fact, 14 mothers described they cried constantly, did not eat, or were depressed because of their children's absence. Mothers also expressed feeling guilty about not being able to be with their children.
Dreby (38)	Mexico	USA	*N* = 136 parents	N/A	N/A	Mothers described leaving their children was a difficult decision filled with sadness and inability to concentrate. Some mothers describe even having difficulty to sleep. It was also found that mothers are expected to provide more emotional support and maintain an emotional connection to her children in the country of origin, compared to fathers.
Hondagneu-Sotelo et al. (5)	Mexico, El Salvador, and Guatemala^*^	USA	*N* = 26	N/A	N/A	Participants described experiencing pain and sadness about not being able to live with their children and about having to negotiate their transnational relationships.
Horton (28)	El Salvador	USA	*N* = 12	50% had been in the US for less than 5 years	N/A	Participants described feelings of grief, pain, sadness, worry, and depression stemming from being separated from not being able to be with their children.
Madianou (39)	Philippines	UK	*N* = 52	54% arrived to the UK between 1973 and 1995. Forty-six percent arrived after 2000	*M* = 15.65 years	Participants described feeling sad, crying, having emotional pain, and feelings of failure by not being able to be and/or reunite with their children.
Parreñas (40)	Philippines	Italy and USA	*N* = 72	N/A	N/A	Participants described feelings of helplessness, loneliness, pain, anxiety, loss, and guilt. This emotional distress is exacerbated by taking care of children of their own as domestic workers.
Schmalzbauer (41)	Honduras	USA	*N* = 157	N/A	N/A	Participants described feeling great distress, emotional burden, and feeling heartbroken about being separated from their children, particularly from younger children. Parents feared that young children would not be able to remember them.

*N/A indicates information was not available*.

* *Percentage breakdown was not provided*.

We also assessed the quality of quantitative studies using the Quality Assessment Tool for Observational Cohort and Cross-sectional Studies from the National Heart, Lung, and Blood Institute. This 14-item tool is designed to help reviewers assess elements of rigor and guide a qualitative risk of bias assessment as low, moderate, or high. Some of the items to assess the rigor include the population assessed in the study, justification about the sample size, the use of follow-up measurements, among others. Using this tool, two researchers independently assessed the risk of bias of all six quantitative studies and compared their ratings (Supplementary Table 2). Disagreement about the ratings was discussed until an agreement was reached.

## Results

We identified 17 studies that met the selection criteria. Thirteen of the 17 studies included focused exclusively on Latina women. Of the 17 studies, six were quantitative, eight were qualitative, and three were mixed-methods. The six quantitative studies discussed symptoms of depression among transnational mothers. Of these six quantitative studies, three found a positive association between transnational motherhood and depressive symptoms and three of them did not find an association. The qualitative findings of the three mixed-methods studies discussed the emotional hardships faced by transnational mothers, but only one of them included quantitative information on depressive symptoms among transnational mothers. The eight qualitative studies identified also highlighted the significant emotional distress transnational mothers experience.

### Quantitative Studies

Literature on transnational motherhood is rather scarce, especially regarding their mental health and emotional distress. Most quantitative studies have come out within the last 15 years. Four (16, 30, 31, 33) of the six studies focused on women who had migrated from Latin American countries to the United Sates. Most quantitative studies included recent immigrant women who had moved to the host country within the last 10 years (16, 29–31). The average age of the mothers ranged from 28 (10) to 39 years (32). Most women included in the studies had less than 12 years of education (16, 29, 31). The studies that provided information on the average length of separation between the mother and the children (30, 31, 33) ranged from 3 years (31) to about 6 years (30).

Miranda et al. (16) conducted the first quantitative study investigating the prevalence of depression among mothers who had a child living in the country of origin. In the study, the researchers screened 5,122 Latina immigrant mothers for major depression using the Primary Care Evaluation of Mental Disorders (Prime-MD). The study found that Latina mothers who had a child living in their country of origin, were 1.52 times more likely to experience depression compared to Latina immigrant mothers whose children lived with them and compared to Latina immigrant women who did not have any children (10). The risk of bias assessment indicated that this study had a low risk. One of the main strengths of this study is that it included a large and diverse sample of women from several countries in Central and South America and it was the first study to quantitatively assess the prevalence of depression among transnational mothers. However, the main limitation is that all women in the study were receiving services for a US-born child, which excluded mothers who did not have any children in the host country or were not receiving services for them.

More recent studies have found similar results. Ornelas and Perreira (31) studied the mental health outcomes of 281 Latina women living in the United States, with 41% having been separated from their children when migrating to the United States. Of the women who had been separated from their children, the average length of separation was 3 years. The researchers used the Patient Health Questionnaire (PHQ-9) and the Center for Epidemiological Studies Depression Scale (CES-D) to assess depressive symptoms. The study found that reunification with children reduced parents' risk for depressive symptoms, which supports the notion that separation may increase mothers' risk for depression (31). The risk of bias assessment suggested that this study had a low risk. One of the main strengths of this study is that it was conducted in North Carolina, which is considered to be a new destination for Latinx immigrants (31). However, one of the main limitations of the study is that participant recruitment was done through schools, which limited the pool to families who had already reunited, but could have been previously separated.

In a study of 514 immigrant women in Canada, Bouris and colleagues showed that 92 mothers had been separated from a child due to international migration (29). Researchers measured depressive symptomatology using the Edinburgh Postnatal Depression Scale and found that 28.3% of separated mothers had elevated depressive symptoms, compared to 18.6% of non-separated mothers, a difference that was statistically significant (29). Aside from postpartum depressive symptoms, researchers also found that separated mothers were about twice as likely to be *clinically* depressed than non-separated mothers (23.1 vs. 13.5%), as measured using parts one and two of the Hopkins Symptom Checklist-25 (29). The risk of bias assessment indicated that this study had a low risk. One of the main limitations of this study is that mothers were recruited from birthing units. It is possible that having a child in the host country could exacerbate depressive feelings among mothers by reminding them of their children and their experiences of motherhood in the country of origin.

However, not all quantitative studies have found a positive association between transnational motherhood and maternal depressive symptoms. In a study of Mexican immigrant parents who had been separated from their children, Rusch and Reyes (33) analyzed depressive symptom scores using the Spanish version of the CES-D for 37 parents who had been separated, and they compared the scores with scores from 16 non-separated parents. The study found that separated parents did not experience higher levels of depressive symptoms than non-separated parents. However, 43% of parents in the study scored 16 or above in the CES-D, indicating elevated depressive symptoms in the overall sample (33). The risk of bias assessment for this study suggested a moderate risk. A particular strength of this study is that it included participants from a community sample, increasing diversity among participants. However, this study had a small sample size, particularly of non-separated parents (*n* = 16), which might not be sufficiently powered to detect significant differences.

In a study of Latina women, McCabe et al. (30) found that separations were not directly related to depressive symptoms. In this study, researchers compared depressive symptom scores measured using the CES-D among 60 women with separations from an adult child, 37 women with separations from minor children, and 328 women with no separation from children (30). A comparison of scores revealed that separation was not directly associated with depressive symptoms (30). However, in this same study, researchers found that separation can lead to economic and immigration-related stress, which in turn can increase the risk for mental health issues (30). The risk of bias assessment for this study suggested a moderate risk. Among its strengths, this study assessed whether the age (adult vs. minor) of the children left behind impacted maternal depressive symptoms differently. However, it is possible that the sample size of the different groups, particularly of adult children (*n* = 37) was too small to detect significant differences.

Lastly, in a study of 2,468 Sub-Saharan African migrants residing in France, Pannetier et al. (32) measured anxiety and depressive symptoms using the PHQ-4. The study found that cross-border separation from a minor child (i.e., less than 18 years of age) was not directly associated with anxiety or depressive symptoms (32). Assessment of the risk of bias indicated that this study had a low risk. This study had a large sample size, which contributed to its strengths. However, this study used the PHQ-4, which is a tool that has not been validated among diverse populations from Africa.

### Mixed-Methods Studies

The three mixed-methods studies identified (34–36) support a potential association between transnational motherhood and emotional distress. Two of the studies were conducted in the United States (35, 36). All three studies were highly diverse in their samples, which included participants originating from different countries around the world (e.g., China, Haiti, Mexico). Most mothers included in the studies had less than a high school education (34–36). The studies found that the average length of separation ranged from 1.2 years (35) to 3.3 years (34). However, the studies by Suárez-Orozco et al. (35, 36) suggest that the length of separation depended on the family's country of origin. For instance, mothers from Mexico and China reunited with their children faster than families from Central America and Haiti (35, 36).

One mixed-methods study of 113 refugees in Canada from Latin America and Africa found that separation from children was a traumatic event that contributed to psychological distress among parents (34). In this study, researchers used the Symptom Checklist (SCL-90) to measure several mental health symptoms among fathers and mothers. The results indicated that there was not a statistically significant difference on mental health symptoms based on reunification patterns with their children. Researchers also conducted semi-structured interviews with 113 parents, of whom 70% had not yet reunited with their children after an average of 3.5 years (34). A qualitative analysis revealed feelings of powerlessness, worry, and an overall sense of hopelessness among participants who were or who had been separated from children (34). Although this study provided robust information regarding mental health symptoms among refugee populations in Canada, it is possible that the sample sizes for the quantitative analysis were too small to identify a significant difference across different reunification patterns. Moreover, this study did not provide a stratified analysis by sex on the SCL-90, which could have provided different results.

The second mixed-methods study identified in the review consisted of 309 Chinese, Central American, Dominican, Haitian, and Mexican parents who had been separated from their children at some point during the migration journey (35). The researchers used a 26-item psychological symptom scale, which was informed by the fourth edition of the Diagnostic and Statistical Manual of Mental Disorders and the SCL-90 (35). Unfortunately, the quantitative results are only available for children and not for parents, thus they are not reported here. This study also collected qualitative data from the parents and it found that mothers described feelings of agony and sadness after separating from their children (35). The last mixed-methods study was conducted by Suárez-Orozco et al. (36) with 385 transnational families. Similar to the previous study (36), the quantitative data was only provided for adolescents. The qualitative data collected among parents found that immigrant parents consistently described feelings of sadness stemming from separating from their children. In this study, qualitative interviews revealed poignant feelings of sadness and desperation among parents during separation (36). These two studies provided important and relevant information about the repercussions that separations had for the family. Although data were collected from adolescents and parents, the results focused more on the adolescents than the parents.

### Qualitative Studies

Eight of the 17 studies identified were qualitative. Although inferences about correlations or associations cannot be made from qualitative studies, they do provide important information about the lived experiences of transnational mothers who have at least one child in their country of origin. Most studies were conducted in the United States (5 out 8), one was conducted in Canada (37), one in the United Kingdom (39), and one in Italy and the United States (40). Six studies focused on the experiences of women from Latin American countries (5, 11, 28, 37, 38, 41) and the other two focused on women from the Philippines (39, 40). Most studies indicated that the majority of their participants left their children under the care of grandparents in their country of origin, usually maternal grandparents. Only three studies (11, 37, 39) provided information regarding the length of separation between the mothers and their children, which ranged from 2 months (37) to an average of 15.6 years (39).

The majority of the studies we found did not focus exclusively on emotional distress or depressive symptoms, but these experiences became evident throughout the descriptions the participants provided. It is important to underscore that many of the experiences described by transnational mothers fall under the criteria of Major Depressive Disorder (MDD), which include depressed mood, loss of interest, excessive guilt, weight loss/gain, loss of energy, insomnia, inability to concentrate, and thoughts of death (42). These criteria are not to say that if assessed, transnational mothers would have a formal MDD diagnosis; however, it provides information regarding how their feelings may indicate that some depressive symptomatology may be present.

In the qualitative literature, many participants described being constantly sad (5, 11, 37) and even depressed (11, 28, 37). In many studies, participants mentioned crying very often, especially when they think about their children and also when they talk to them over the phone (11, 38–40). For many mothers, the hardest part of migrating to another country has been leaving their children behind (11, 38), particularly if their children were very young when they left (41). Studies also described that many mothers felt pain and guilt for leaving their children in their country of origin, being accused of not being good mothers, and for not giving them personal love and attention (5, 11, 39–41). Although most transnational mothers hope the reunification process is fast, many end up being separated from their children for many years (39, 40), even to the point where they can no longer reunite because children are over 18 (37). These long-term separations appeared to exacerbate feelings of helplessness and hopelessness because mothers were unsure about when they would be able to see their children again (37, 40). Sometimes these feelings can be ameliorated based on the type of childcare arrangements that mothers had for their children in the country of origin. Most studies (5, 11, 37–40) reported that maternal grandparents or female relatives were the ones in charge of taking care of the children after the mother migrated, which made mothers feel comfortable because they knew their children were being cared for.

Not only did some mothers describe emotional feelings, but they also described physical ones (11). Dreby (38) found that some mothers described losing weight and even becoming physically ill after migrating to the United States and leaving their children in their country of origin. Some studies have also documented mothers describing not being able to sleep thinking about their children and the conditions they might be living under; they wonder whether the conditions have changed at all since they left and whether their sacrifice has been worthwhile (21, 29, 38). A couple of studies (29, 41) found that mothers have a sense of loss and grief after leaving their children in their country of origin. Although not very common, one study (37) found that 22% of women were so depressed they had difficulty carrying out their daily activities and 10% of women did not find meaning in their lives without their children. Overall, findings from qualitative studies demonstrate that transnational motherhood may exacerbate feelings of sadness and guilt, particularly during the first years after migrating to a new country.

## Discussion

This scoping review of 17 quantitative, qualitative and mixed-methods studies indicates that transnational mothers experience emotional distress when they move to a host country and have to leave their children in their countries of origin. This emotional distress among transnational mothers ranges from being sad to experiencing depressive symptoms that interfere with their daily tasks. However, from the six quantitative studies found and reviewed, it is not possible to unequivocally argue that transnational motherhood is associated with higher symptoms of depression. Out of these 6 studies, three demonstrated that transnational mothers had higher levels of depressive symptoms (16, 29, 31) and three did not find a statistically significant association (30, 32, 33). It is important to highlight that these studies were heterogenous in terms of recruitment approaches (e.g., birthing units, health care settings, schools), measurement tools (e.g., CESD, PHQ-4, PHQ-9), and sample sizes, which contributed to mixed results.

Findings from mixed-methods and qualitative studies found that transnational mothers are negatively impacted by the separation from their children. Feelings of emotional distress included descriptions of constant sadness, crying spells, hopelessness, helplessness, guilt, difficulty sleeping, sense of loss, and depression. Taken together, the findings from this scoping review suggest that transnational mothers struggle emotionally when they leave their children in their country of origin, but the literature, particularly quantitative, is still in its infancy to make definitive conclusions regarding the relationship between transnational motherhood and depressive symptoms. Future epidemiological studies should include an assessment of transnational motherhood in order to have a better understanding of its prevalence of and how the length of separation and the reunification process impact family dynamics in the long-term. Having a better understanding of the prevalence of transnational motherhood would constitute the first step in determining ways to identify, reach and engage transnational mothers, if deemed appropriate.

Future studies should also investigate moderating factors that may exacerbate or mitigate the effect of transnational motherhood on depressive symptoms. For instance, it would be important to determine whether immigration status and proximity to the country of origin could exacerbate or ameliorate depressive symptoms and emotional distress. Immigration status is one of the most important constructs to understand in transnational motherhood given that depending on the status of the mother, the process of reunification with her children in the host country could be faster or slower (40). Proximity to country is yet another important construct to investigate because mothers that live relatively closer to their children (e.g., United States and Mexico) might be able to interact with them more and/or more often given that they would not have to worry about issues such as having different time zones (39). Having constant communication would in turn help them cope with the separation more effectively (5, 40).

As more research continues advancing the field of transnational motherhood, it is necessary to support transnational mothers and their families in the meantime. One way to support them is by identifying the coping mechanisms they use to deal with their emotional distress. For instance, many studies have shown that sending remittances back to the country of origin sparks feelings of happiness and fulfillment (28). Being able to send gifts and remittances to their children makes women feel empowered and it reminds them that their sacrifice has been worth the effort of separating from their children (5, 28, 40, 41). Recent research on remittances among Latinx immigrants further suggests that sending remittances to the home country is associated with lower odds of depression and psychological distress (43, 44). Specifically, these studies found a 20% reduction in the odds of depression (45) and 19% decrease in the odds of psychological distress (44) among Latin American immigrants who sent more remittances.

Another coping mechanism that transnational mothers use to feel close and connected to their children is having constant communication with them (5, 40). Facilitating communication between transnational mothers and their children is through the use of information communication technologies (ICTs) such as phones, computers, and video-calls (39, 46). Previous research has demonstrated that transnational families remain in contact and even feel close with one other when using ICTs (39, 46). A qualitative study of 52 Filipina mothers living in the United Kingdom found that through the use of ICTs, transnational mothers were able to monitor their children from a distance by having routine video calls every morning (29). Also, ICTs have increased communication levels among transnational families since consistent interaction makes it more difficult for those living abroad to hide the difficulties they are facing (39). This constant interaction and even the conflict that transnational families encounter may make the experience of being a mother more “realistic” (39). ICTs have also been used as tools in family therapy (46). For instance, Bacigalupe and Lambe (46) describe the way in which they used ICTs in family therapy to treat behavioral problems among Guatemalan children who had recently migrated to the US. From the assessment, it was clear that the children missed their aunt and grandmother, who were still in Guatemala, and who had raised the children until they were brought to the US (46). The family then started meeting through video calls and issues of school conflict and expectations about school were addressed with all the family members (46). The aforementioned studies demonstrate that it can be possible and effective to include ICTs when working with transnational families.

Overall, this scoping review demonstrates there is qualitative evidence suggesting there is a link between transnational motherhood and depressive symptoms and emotional distress; however, there is a paucity of quantitative research to supports this evidence. In order to promote this research area, it is important to increase *interdisciplinary research* on the lives of mothers and families. Researchers in the social sciences engage with conceptualizations of depressive symptoms as described by women participants in their qualitative studies and these are not always part of epidemiological studies. There is a need to work outside the boundaries of disciplines in order to fully grasp how depression and other mental health concerns are discussed across cultures. In depth interviews and ethnographic approaches are valuable tools to further understand how women make sense of motherhood, womanhood and their role as caregivers in both their countries of origin and the host country. Also, studies that make connections across borders may be revealing in terms of how mental health is discussed and defined “here” and “there.” These studies could potentially identify the emotional attachments that transcend national borders, which in turn could inform the development of culturally appropriate measurement tools that can accurately identify depressive symptoms and other mental health symptoms among transnational mothers.

## Strengths and Limitations

This study has a number of strengths that should be considered. First, to our knowledge this is the first study focusing exclusively on summarizing the literature available on depressive symptoms and emotional distress among transnational mothers. This review follows a systematized process that ensures replicability, as well as the inclusion of quantitative, qualitative, and mixed-methods studies that capture the breadth and depth of the current literature available in the field. Nevertheless, this study also has some limitations. First, this study did not include book chapters or dissertations available in the topic of transnational motherhood, which limited the amount of information included and reviewed. Second, this study mostly focused on depressive symptoms; however, there are other conditions, such as anxiety, post-traumatic stress disorder, and alcohol misuse, which usually coexist with the aforementioned symptoms and should be investigated. Finally, this study predominantly focused on the experiences of mothers given that there is a very limited amount of research conducted on transnational motherhood. However, it is necessary that future studies focus on understanding the emotional sequelae that paternal separation has on fathers. Some of the studies included in this review also included the experience of fathers (11, 31–36, 38), and they suggest that fathers also experience emotional distress, which may co-occur with alcohol misuse (35, 36).

## Conclusion

Despite the scarce amount of research available on the mental health needs of transnational mothers, the current literature seems to suggest that transnational mothers experience significant emotional distress after migrating to host countries. Results from quantitative studies provide inconclusive information regarding the association between depressive symptoms and transnational motherhood. Findings from mixed-methods and qualitative studies indicate that transnational mothers experience emotional distress that is consistent with symptoms of major depression. The paucity of literature on the mental health needs of transnational mothers indicates the critical need to continue investigating and identifying their needs.

## Author Contributions

MP-L conceived the study, conducted the initial article search, screened full articles, and drafted the manuscript. LY helped conducting the study, screening the titles, abstracts and full articles, contributed to writing the methods and the results section, and provided in-depth feedback on the manuscript. AY contributed to writing the introduction and organizing the results section. GO identified additional studies as an expert in the topic, contributed to the discussion section, and provided in-depth feedback on the manuscript. All authors discussed the results and approved the final manuscript.

## Conflict of Interest

The authors declare that the research was conducted in the absence of any commercial or financial relationships that could be construed as a potential conflict of interest.
