# Supplementary material for: Depressive Symptoms and Emotional Distress of Transnational Mothers: A Scoping Review
Source: Front Psychiatry. 2021 Feb 25;12:574100. doi: 10.3389/fpsyt.2021.574100 (PMC7947237; doi:10.3389/fpsyt.2021.574100)
Supplement: Supplementary file 1 [file Table_1.DOCX]

| Supplemental table 1. Search strategy | | | |
| --- | --- | --- | --- |
| Database | Keywords/terms searched | Number of articles identified | Date searched |
| PubMed | (women OR migrant OR immigrant OR transnational OR transnational mother OR immigrant mothers OR migrant mothers OR transnational mothers) AND (mood disorders OR emotional distress OR depression) | 186 | 6/18/2019 |
| PsycINFO | (women OR migrant OR immigrant OR transnational OR transnational mother OR immigrant mothers OR migrant mothers OR transnational mothers) AND (mood disorders OR emotional distress OR depression) | 241 | 6/18/2019 |
| ERIC | (women OR migrant OR immigrant OR transnational OR transnational mother OR immigrant mothers OR migrant mothers OR transnational mothers) AND (mood disorders OR emotional distress OR depression) | 3,397 | 6/18/2019 |
| CENTRAL | (women OR migrant OR immigrant OR transnational OR transnational mother OR immigrant mothers OR migrant mothers OR transnational mothers) AND (mood disorders OR emotional distress OR depression) | 17 | 6/18/2019 |
| Scopus | (women OR migrant OR immigrant OR transnational OR transnational mother OR immigrant mothers OR migrant mothers OR transnational mothers) AND (mood disorders OR emotional distress OR depression) | 75 | 6/18/2019 |
| ScienceDirect | (women OR migrant OR immigrant OR transnational OR transnational mother OR immigrant mothers OR migrant mothers OR transnational mothers) AND (mood disorders OR emotional distress OR depression) | 4,463 | 6/23/2019 |
| Total |  | 8,379 |  |
